# HK2 is associated with the Warburg effect and proliferation in liver cancer: Targets for effective therapy with glycyrrhizin

**DOI:** 10.3892/mmr.2021.12143

**Published:** 2021-05-10

**Authors:** Zengpeng Sun, Zhiguo Tan, Chuang Peng, Weimin Yi

Mol Med Rep 23: 343, 2021; DOI: 10.3892/mmr.2021.11982

Following the publication of the above article, the authors have realized that the Funding section of the Declarations on p. 7 was written incorrectly: The text here should have appeared as follows: ‘This work was supported by the Research Fund Subsidy Project of Hunan Provincial Committee on Health Commission (grant no. 20200179) and Xiang Wei Medical Administration Medical Office Memorandum (2019) No. 118 and the Research Fund of Hunan Provincial Department of Education (grant no. 20C1163)’.

The authors regret their oversight in providing this incorrect information in the Funding section of their paper. They thank the Editor of *Molecular Medicine Reports* for allowing them the opportunity to publish this corrigendum, and apologize to the readership of the Journal and to the inappropriately credited funding bodies in question for any inconvenience caused.

